# Impact of combined management strategies of monensin and virginiamycin in high energy diets on ruminal fermentation and nutrients utilization

**DOI:** 10.3389/fvets.2024.1325198

**Published:** 2024-03-28

**Authors:** João V. T. Dellaqua, André L. N. Rigueiro, Antonio M. Silvestre, Murilo C. S. Pereira, Luana D. Felizari, Breno L. Demartini, Evandro F. F. Dias, Leandro A. F. Silva, Daniel M. Casali, Katia L. R. Souza, Johnny M. Souza, Danilo D. Millen

**Affiliations:** ^1^Department of Breeding and Animal Nutrition, School of Veterinary Medicine and Animal Science, São Paulo State University (UNESP), Botucatu, Brazil; ^2^Department of Animal Production, School of Agricultural and Technological Sciences, São Paulo State University (UNESP), Dracena, Brazil; ^3^Department of Animal Science, School of Agricultural and Veterinary Studies, São Paulo State University (UNESP), Jaboticabal, Brazil

**Keywords:** feed additive, feedlot, ionophore, metabolism, rumen

## Abstract

Feed additives such as monensin (MON) and virginiamycin (VM) are commonly utilized in feedlot diets to enhance rumen fermentation. Nevertheless, the precise effects of combining MON and VM during specific feedlot periods and the advantages of this combination remain unclear. This study was designed to investigate the effects of withdrawal of MON when associated with VM during the adaptation and finishing periods on ruminal metabolism, feeding behavior, and nutrient digestibility in Nellore cattle. The experimental design was a 5 × 5 Latin square, where each period lasted 28 days. Five rumen-cannulated Nellore yearling bulls were used (414,86 ± 21,71 kg of body weight), which were assigned to five treatments: (1) MON during the entire feeding period; (2) VM during the entire feeding period; (3) MON + VM during the adaptation period and only VM during the finishing period 1 and 2; (4) MON + VM during the entire feeding period; (5) MON + VM during the adaptation and finishing period 1 and only VM during the finishing period 2. For the finishing period 1, animals fed T3 had improved potential degradability of dry matter (*p* = 0.02). Cattle fed T3 and T5 had the highest crude protein degradability when compared to animals receiving T2 (*p* = 0.01). Animals fed T2 and T3 had reduced the time (*p* < 0.01) and area under pH 6.2 (*p* = 0.02). Moreover, animals fed T4 had greater population of protozoa from the genus *Diplodinium* (*p* = 0.04) when compared to those from animals fed T2, T3 and T5. For the finishing period 2, animals fed T3 had greater starch degradability when compared to animals receiving T4 and T5 (*p* = 0.04). Animals fed T3, T4 and T5 had increased the duration of time in which pH was below 5.6 (*p* = 0.03). The area under the curve for ruminal pH 5.2 and pH 5.6 was higher for the animals fed T3 (*p* = 0.01), and the area under pH 6.2 was higher for the animals fed T3 and T5 (*p* < 0.01) when compared to animals receiving T2. There is no substantial improvement on the rumen fermentation parameters by the concurrent utilization of MON and VM molecules, where the higher starch and protein degradability did not improve the rumen fermentation.

## 1 Introduction

The inclusion of higher energy levels in feedlot animal diets can lead to various digestive disorders (1) due to the high fermentability of the ingredients used, often involving grains and concentrate feedstuffs (2, 3). These are incorporated to maintain production efficiency within economically viable limits. Consequently, optimizing animal weight gain can be achieved through either increasing dry matter intake (DMI) or enhancing animal efficiency.

In line with the fermentative potential of diets, the use of feed additives that can improve feed efficiency, weight gain, and even have positive environmental impacts by reducing methane emissions, has gained traction among nutritionists. Among these additives, ionophores are extensively utilized in feedlots in Brazil and North America (2, 3). Their mode of action is well-established; they target gram-positive bacteria, which alters the fermentation process in the rumen, resulting in elevated propionate levels and decreased acetate levels (4).

Sodium monensin (MON), a carboxylic polyether ionophore (5), has been extensively researched for its impact on feedlot cattle performance (6). A meta-analysis by Duffield et al. (7) revealed a reduction in DMI by 3.1% and an improvement in gain-to-feed (G:F) ratio by 6.4% in feedlot cattle fed MON. Furthermore, combinations of ionophores and antibiotics have been explored (8–11). One commonly associated antibiotic is virginiamycin (VM), a non-ionophore antibiotic that hinders protein synthesis by attaching to the 50S ribosomal subunit (12) and inhibits the growth of Gram-positive bacteria, especially lactic acid-producing bacteria (13). Virginiamycin may also exhibit a protein-sparing effect (14), reducing ruminal protein degradation (15, 16) and enhancing post-ruminal nutrient uptake (17). These effects imply better gains for animals, thanks to increased gain efficiency and dietary net energy for maintenance (NEm) and gain (NEg) (9, 18, 19). Salinas-Chavira et al. (20) and Salinas-Chavira et al. (14) observed an enhancement in G:F ratio by 3.83 and 4.20%, respectively, with the inclusion of VM in the diets of Holstein cattle in feedlots. These additives improved the G:F ratio when administered independently in feedlot cattle; however, only MON has demonstrated a reduction in DMI (7). Additionally, Rigueiro et al. (10) demonstrated in a performance study that the removal of MON increased DMI, resulting in improved animal weight gain.

Research on the combined effects of MON and VM in cattle is limited, and the benefits of this combination remain unclear. Erasmus et al. (17) reported a complementary effect between MON and VM when both were included in the diets of early lactation cows. Previous studies by Nuñez et al. (21) and Lemos et al. (22) explored combinations of MON, salinomycin, VM, and flavomycin, yielding no positive effects on feedlot performance. Nuñez et al. (21) reported that the combination of VM and salinomycin decreased DMI, increased NEm and Neg, suggesting a positive effect of this combination compared to salinomycin; however, no effect on ADG and G:F ratio was observed. Despite these studies evaluating the combination of MON and VM in feedlot diets, none of them specifically addressed the combination of two feed additives in specifics periods. In this context, the present study forms part of a larger research effort by this research group, evaluating the effects of different combinations of MON and VM at specific feedlot periods (10, 11, 23, 24). Rigueiro et al. (10) and Squizatti et al. (24) investigated various combinations of MON and VM throughout the adaptation and finishing stages of feedlot Nellore cattle. Their research revealed that Nellore yearling bulls experienced improved feedlot performance (10) and rumen fermentation (24) with high-concentrate diets containing MON and VM during adaptation, and VM alone during finishing. Moreover, Rigueiro et al. (10) observed enhanced performance in the last 40 days of the study in cattle solely fed VM during the finishing period. Based on these findings, we hypothesized that the withdrawal of MON, when combined with VM and a higher-energy diet during the final third of the feedlot period, would enhance DMI and improve ruminal fermentation and nutrient digestibility. Subsequently, Rigueiro et al. (23) conducted a performance trail and found that withdrawing MON in conjunction with VM, alongside a higher energy diet, during the last 40 days of the feedlot period led to enhanced final body weight (BW), average daily gain (ADG) and hot carcass weight (HCW) in Nellore cattle compared to those fed either MON or VM alone.

Consequently, it was hypothesized that the withdrawal of MON, when associated with VM, combined with a higher energy diet during the final third of the feedlot period would enhance ruminal fermentation and nutrient digestibility in Nellore cattle. Therefore, the present study encompasses the metabolism assay linked with the performance trial conducted by Rigueiro et al. (23). Thus, this study was designed to evaluate the effects of withdrawal of MON when associated with VM during the adaptation and finishing periods on ruminal metabolism, feeding behavior, and nutrient digestibility in Nellore cattle.

## 2 Materials and methods

The protocols and procedures followed in this study were approved by the São Paulo State University Ethical Committee for Animal Research (protocol number CEUA - 154/2016). The study was conducted at the São Paulo State University feedlot, Dracena campus, Brazil.

### 2.1 Animals and treatments

Five rumen-cannulated Nellore yearling bulls (36-mo-old, 414,86 ± 21,71 kg) were randomly assigned to a 5 × 5 Latin square design. Each period lasted 28 days, 14 days of adaptation diets and 14 days of the finishing diet, and the cattle were randomly assigned to different treatment.

The experimental treatments were as follows: T1) MON during the entire feeding period; T2) VM during the entire feeding period; T3) MON + VM during the adaptation period and only VM during the finishing period 1 and 2; T4) MON + VM during the entire feeding period; T5) MON + VM during the adaptation and finishing period 1 and only VM during the finishing period 2. Doses used in this study were based on Rigueiro et al. (10, 23) when either MON (30 mg/kg of DM;) or VM (25 mg/kg of DM) were fed as sole feed additives in the diet. MON (Bovensin 200; Phibro Animal Health Corporation, Guarulhos, São Paulo, Brazil) was added at 1000 mg/kg of mineral mixture and VM (V-Max 2; Phibro Animal Health Corporation, Guarulhos, São Paulo, Brazil) was added at 833 mg/kg of mineral mixture and offered to yearling bulls according to the treatments.

### 2.2 Feeding and management description

At the beginning of the study, all steers underwent deworming and vaccination (tetanus, bovine viral diarrhea virus, 7-way *Clostridium* sp.; Cattlemaster and Bovishield, Pfizer Animal Health, New York, NY, United States). Subsequently, Nellore steers were individually housed in pens measuring 72m^2^ each, with 6 meters of linear bunk space provided and free access to water via a shared drinking fountain (3.00 × 0.80 × 0.20 m) for two animals.

Animals were provided *ad libitum* feed once daily at 0800 h, and DMI was calculated by weighing the ration offered and leftovers before the next morning delivery, expressed both in kilograms and as a percentage of BW. Dietary DM content was determined daily following method 934.01 (25). The amount of feed offered was adjusted daily based on the targeted quantity of orts (3 to 5%) remaining before the morning feed delivery (0700 h). The BW was measured at the beginning (day 1) and at the end (day 28) of each period at 0700 h.

Experimental diets were formulated according to the Large Ruminant Nutrition System (26) and are shown in Table 1. The step-up adaptation program involved *ad libitum* intake with progressively increasing levels of concentrate ingredients until reaching the concentrate levels of finishing diets 1 (84%) and finishing 2 (88%). The adaptation protocol lasted 14 days, during which three adaptation diets containing 66, 72 and 78% concentrate were fed for 5, 4 and 5 days, respectively.

**Table 1:** Feed ingredients and chemical composition of high-concentrate diets fed to Nellore yearling bulls during adaptation and finishing periods.

Item	Percent of concentrate^1^				
	66	72	78	84	88
*Ingredients, % of DM^2^*					
Sugarcane bagasse	14.00	14.00	14.00	14.00	10.00
*Cynodon dactylon* hay	20.00	14.00	8.00	2.00	2.00
Finely ground corn grain	46.00	54.00	62.00	70.00	76.70
Soybean meal	17.30	15.10	12.90	10.70	8.00
Mineral mixture^3^	2.30	2.30	2.40	2.40	2.40
Urea	0.40	0.60	0.70	0.90	0.90
*Nutrient content, % of DM^4^*					
DM, as % of organic matter	74.00	74.00	73.00	73.00	74.00
Total digestible nutrients	72.00	72.00	75.00	78.00	80.00
Crude protein	15.20	15.00	14.60	14.50	14.00
Neutral detergent fiber	34.30	30.50	26.80	23.00	19.20
Non-fiber carbohydrates	43.00	48.00	52.00	57.00	61.00
peNDF ^5^	26.00	22.00	18.00	14.00	10.00
Neg ^6^, Mcal/kg	1.09	1.09	1.15	1.25	1.29
Ca	0.60	0.58	0.56	0.54	0.52
P	0.40	0.41	0.42	0.42	0.42

^1^Adaptation diets (66 to 78%), and finishing 1 (84%) and 2 (88%) diets. ^2^Dry matter. ^3^Mineral mixture contained: Ca: 182 g/kg of DM; P: 40.5 g/kg of DM; Mg: 7.7 g/kg of DM; K: 0.5 g/kg of DM; Na: 82.2 g/kg of DM; Cl: 126.5 g/kg of DM; S: 16 g/kg of DM; Co: 27.50 mg/kg of DM; Cu: 754.17 mg/kg of DM; Fe: 2498 mg/kg of DM; I: 37.29 mg/kg of DM; Mn: 740 mg/kg of DM; Se: 6.20 mg/kg of DM; Zn: 1790 mg/kg of DM. Monensin was added at 1000 mg/kg of mineral mixture and Virginiamycin was added at 833 mg/kg of mineral mixture. ^4^Estimated by equations according to Large Ruminant Nutrition System (26). ^5^Phisically effective NDF, determined according to the method described by Heinrichs and Kononoff (27). ^6^Net energy for gain, expressed in Mcal per kg of diet DM.

### 2.3 *In situ* degradability

The *in situ* degradability determination was carried out following a methodology adapted from Mehres and Ørskov (28) on days 16, 17, 18, and 19 for finishing diet 1, and 23, 24, 25 and 26 for finishing diet 2. Approximately 15 g of diet samples, previously dried at 65°C for 72 h, were placed into nylon bags with a porosity of 50 microns (dimensions: 10.0 × 19.0 cm) in triplicate. These bags were then introduced into the rumen and incubated for 3, 6, 12, 24, 48, and 72 h. Following retrieval from the rumen, the nylon bags were washed with cold running water and subsequently oven-dried at 65°C for 72 h. Samples that were not incubated in the rumen underwent the same washing procedure.

Samples were analyzed for crude protein (25) by total N determination using the micro-Kjeldahl technique (method 920.87; (29)); NDF, with heat-stable α-amylase (30); and starch (31). Potential degradation and effective degradability of DM, starch, and crude protein were calculated according to Ørskov and McDonald (32), and effective degradability was estimated for each ingredient assuming the rumen solid outflow rates of 0.02, 0.05, and 0.08 h^−1^, which are representative of low, medium and high feeding amounts (33).

### 2.4 Feeding behavior and particle sorting

Cattle underwent visual observations to assess feeding behavior at 5-min intervals throughout a 24-h period on days 11, 17, and 24 of each experimental phase. These days corresponded to the adaptation, finishing period 1, and finishing period 2, respectively. The visual observations adhered to the methodology outlined by Robles et al. (34). Feeding behavior data were recorded for each individual animal as follows: time spent eating, ruminating, and resting (expressed in minutes), as well as the frequency of meals per day. A meal was defined as the continuous period during which cattle consumed the ration uninterruptedly from the feed bunk. Meal length in minutes was computed by dividing the total time spent eating by the number of meals per day. The DMI per meal in kilograms was calculated by dividing DMI by the number of meals per day. Additionally, the time spent eating and time spent ruminating data were utilized to calculate the eating rate of DM (ERDM; time spent eating/DMI) and rumination rate of DM (RRDM; time spent ruminating/DMI), both expressed in minutes per kilogram of DM.

On the day when feeding behavior data were gathered, samples of the diet and orts were obtained to evaluate particle-size distribution. The analysis was conducted using sieving techniques with the Penn State Particle Size Separator, and the results were reported based on an as-fed basis following the methodology outlined by Heinrichs and Kononoff (27). Particle sorting was determined as follows: n intake / n predicted intake, in which n = particle fraction retained on screens of 19 mm (long), 8 mm (medium), and 1.18 mm (short) and a pan (fine). Particle sorting values equal to 1 indicate no sorting. Those <1 indicate selective refusal (sorting against), and those>1 indicate preferential consumption (sorting for), according to Leonardi and Armentano (35). Furthermore, samples of the diet and ort were collected on the days of feeding behavior data collection for chemical analysis of neutral detergent fiber (NDF; (30) to determine the intake of NDF. The eating rate of NDF (ERNDF) was then calculated by dividing the time spent eating by NDF intake. Similarly, the rumination rate of NDF (RRNDF) was determined by dividing the time spent ruminating by NDF intake. Both ERNDF and RRNDF were expressed in minutes per kilogram of NDF.

### 2.5 Ruminal fermentation variables

Ruminal pH was continuously measured every 10 min, on days 18, 19, and 20 on finishing periods 1 and days 25, and 26 and 27 on finishing period 2 of each experimental period, using a Lethbridge Research Centre Ruminal pH Measurement System (LRCpH; Dascor Inc., Escondido, CA, United States) in accordance with the methodology outlined by Penner et al. (36). The pH electrode (model T7-1 LRCpH, Dascor, Escondido, CA model S650) was enclosed by a shroud that allowed passage of particle and liquid but prevented direct contact between the pH electrode and the ruminal epithelium surface. The capsule was attached to the ruminal cannula plug to facilitate positioning within the rumen and to maintain the electrode in a vertical orientation. To ensure placement in the ventral sac of the rumen, two 900-g weights were attached to the base of the electrode shroud. Before placing the LRCpH system into the rumen, pH readings were taken in pH buffers 4 and 7. Daily ruminal pH data were averaged and presented as minimum pH, mean pH, and maximum pH, along with metrics such as the area under the curve, and the duration of time in which pH was below 6.2, 6.0, and 5.8. The area under the curve was computed by multiplying the absolute deviations in pH by the time (min) spent below the established threshold for each measure, divided by 60, and expressed as pH unit × hour. Additionally, data loggers recorded rumen temperature and ox-redox potential (36).

During each experimental period, on days 16, 17, 18, and 19 for finishing diet 1, and days 23, 24, 25, and 26 for finishing diet 2, approximately 500 mL of rumen fluid was collected via cannula, at intervals of 0, 4, 8 and 12 h following the morning meal, from three different areas of the rumen. Subsequent to sample collection, the remaining ruminal fluid was promptly returned to the rumen. In the laboratory, the samples were centrifuged at 2,000 × g for 20 min at room temperature, and 2 mL of the supernatant was added to 0.4 mL of formic acid and frozen at −20°C for further short-chain fatty acid (SCFA) analyses, according to Erwin et al. (37). The SCFA concentrations were determined using gas chromatography (Finnigan 9,001, Thermo Scientific, West Palm Beach, FL) equipped with a glass column MEGABOR (model 1 OV-351, Ohio Valley Specialty Chemical, Inc., Marietta, OH, USA) of 1.0 Micron, 1.22 m length and 0.63 cm internal diameter, to determine the concentration of acetate, propionate, and butyrate. Analysis involved injecting 1.0 μL of the sample into the chromatograph, which was linked to a computer running Borwin software (version 1.21) for quantification calculations. Nitrogen served as carrier gas (flow rate: 25 mL/min), hydrogen as fuel gas (15 mL/min) and oxygen as oxidant gas (175 mL/min). Temperatures were set at 220°C for the vaporizer, 250°C for the flame ionization detector, and 195°C for the separation column initially, increasing by 10°C/min until reaching 200°C. A standard solution was used as a reference for organic acid concentrations in the samples. The number of repetitions per sample was adjusted to maintain reading differences below 5%. To mitigate potential column contamination effects on readings, the standard solution was injected after every ten consecutive sample injections. Organic acid concentration calculations were conducted on a computer by comparing samples with the standard solution.

Lactic acid concentration was assessed using a colorimetric method outlined by Pryce (38). To determine NH_3_-N concentration, 2 mL of the supernatant of ruminal samples was mixed to 1 mL of 1 N of H_2_SO_4_ solution, and the centrifuge tubes were promptly frozen until colorimetric analyses were conducted, following the procedure described by Kulasek (39) and adapted by Foldager (40).

### 2.6 Ruminal protozoa counting

To conduct the differential counting of rumen-ciliated protozoa, ruminal contents were manually collected by sweeping the floor of the rumen. Subsequently, 10 mL of this material was preserved in a vial containing 20 mL of 50% (v / v) formaldehyde. Collections were carried out on days 20 and 27 of each experimental period, 4 h after the morning feeding. For analysis, a 1-mL sample was mixed with two drops of 2% brilliant green dye (Sigma, B6756) and diluted with 9 mL of 30% glycerol. Protozoa, including those of the genera *Isotricha*, *Dasytricha*, *Entodinium*, and *Diplodiniinae* subfamily, were identified and counted using a Sedgwick counting chamber Rafter with internal dimensions of 50 mm × 20 mm × 1 mm (capacity 1 mL) through optical microscopy (Olympus CH-2®, Japan) (41).

### 2.7 Ruminal dynamics

On day 28 of each experimental period, the ruminal digesta was completely removed manually from each steer through a rumen cannula, to assess the disappearance rate (Kt) in the rumen, following the methodology described by Dado and Allen (42). The rumen emptying process took place at 11:00, approximately 3 h after the morning meal, under the assumption that the rumen was at its maximum volume. The same procedure was repeated on day 29 of each experimental period, at 8:00, just before the morning meal delivery, assuming the rumen was at its minimum volume at this time. During the process of emptying the ruminal contents, both liquid and solid phases were segregated, weighed, and then a 1 kg sample was homogenized from each steer, considering the proportion of liquid and solid phases, to determine DM. Afterward, rumen digesta was reconstituted and returned to the rumen of the respective steer. The rumen pool of DM and its disappearance rate were calculated based on the dry weight of each sample (55°C for 72 h). The DM disappearance rate was considered equal to the intake rate, and they were estimated using the formula (43): DM disappearance rate (%/h) = Daily DM intake (kg) / DM Ruminal contents (kg) /24.

### 2.8 Statistical analysis

Data were analyzed by PROC MIXED of SAS (SAS Inst., Inc., Cary, NC), where residual normality (Shapiro–Wilk’s and Kolmogorov–Smirnov’s) and variance heterogeneity (GROUP option of SAS) tests were performed before the analysis of variance. In the model, the effect of the treatments was considered fixed; however, the effects of period and animal were considered random factors. Response variables, such as, the molar proportion of SCFA and NH_3_-N concentration were analyzed with repeated measures over time (44), in this case, the model included the same effects just described plus time and its interactions with treatments. Each variable analyzed as repeated measures were subjected to 8 covariance structures: unstructured, compound symmetric, heterogeneous compound symmetric, autoregressive of order one [AR (1)], heterogeneous first-order autoregressive [ARH (1)], Toeplitz, heterogeneous Toeplitz, and ante-dependence of order one [ANTE (1)]. The covariance structure that yielded the smaller Akaike and Schwarz’s Bayesian criterion based on their −2 res log-likelihood was considered to provide the best fit. Differences were considered significant at the *p* ≤ 0.05.

## 3 Results

### 3.1 *In situ* degradability

The results *in situ* degradability of dry matter are presented in Table 2. There was a difference in potential degradability (*p* = 0.02) for the finishing period 1, it shows that the withdrawal of MON after adaptation (T3) improved the degradability when compared to the treatments T2, T4 and T5. In the finishing period 2, there was no difference between the degradability (*p* > 0.05).

**Table 2:** *In situ* degradability of dry matter for rumen cannulated cattle fed high-concentrate diets containing different combinations of virginiamycin (VM) and monensin (MON) during the feedlot period.

Item^2^	Period	Treatments^1^					SEM^2^	*p*-value
	Adaptation:	MON	VM	MONVM	MONVM	MONVM		
	Finishing 1:	MON	VM	VM	MONVM	MONVM		
	Finishing 2:	MON	VM	VM	MONVM	VM		
		(T1)	(T2)	(T3)	(T4)	(T5)		
*Finishing period 1*								
Degradability at 0.02 h^−1^		60.69	61.65	60.66	61.31	60.70	2.16	0.93
Degradability at 0.05 h^−1^		43.75	45.47	43.28	45.27	45.26	2.31	0.63
Degradability at 0.08 h^−1^		35.05	36.67	34.58	36.79	36.85	2.16	0.56
Potential degradability		85.57^ab^	82.94^bc^	87.85^a^	83.62^bc^	81.10^c^	1.73	0.02
*Finishing period 2*								
Degradability at 0.02 h^−1^		64.88	61.83	63.07	63.93	64.53	1.75	0.14
Degradability at 0.05 h^−1^		48.89	45.29	46.43	47.15	48.10	2.14	0.13
Degradability at 0.08 h^−1^		40.05	36.77	37.61	38.43	39.17	2.11	0.19
Potential degradability		85.61	86.61	86.42	87.79	86.37	1.53	0.79

^1^T1 (MON during the entire feeding period); T2 (VM during the entire feeding period); T3 (MON + VM during the adaptation period and only VM during the finishing period 1 and 2); T4 (MON + VM during the entire feeding period); T5 (MON + VM during the adaptation and finishing period 1 and only VM during the finishing period 2). ^2^SEM: standard error of the mean, referent to *n* = 6 pens per treatment; Values within a row with different lower case letters differ (*p* < 0.05).

For starch degradability, there was a difference only for the finishing period 2 (Table 3), for effective degradability at disappearance rates 0.02, 0.05 and 0.08 h^−1^ (*p* = 0.04, *p* = 0.03, p = 0.04 respectively). Animals fed T3 had greater starch degradability when compared to animals receiving T4 and T5; however, there were no differences in starch degradability between T3 and the animals receiving T1 and T2.

**Table 3:** *In situ* degradability of starch for rumen cannulated cattle fed high-concentrate diets containing different combinations of virginiamycin (VM) and monensin (MON) during the feedlot period.

Item^2^	Period	Treatments^1^					SEM^2^	*p*-value
	Adaptation:	MON	VM	MONVM	MONVM	MONVM		
	Finishing 1:	MON	VM	VM	MONVM	MONVM		
	Finishing 2:	MON	VM	VM	MONVM	VM		
		(T1)	(T2)	(T3)	(T4)	(T5)		
*Finishing period 1*								
Degradability at 0.02 h^−1^		77.40	79.85	77.20	77.53	79.33	2.10	0.43
Degradability at 0.05 h^−1^		58.72	62.17	59.01	59.69	62.55	2.87	0.46
Degradability at 0.08 h^−1^		48.30	51.56	48.80	49.46	52.41	2.85	0.51
Potential degradability		99.59	99.11	99.82	99.92	98.72	1.04	0.31
*Finishing period 2*								
Degradability at 0.02 h^−1^		79.92^a^	79.65^a^	80.66^a^	75.43^b^	76.05^b^	1.43	0.04
Degradability at 0.05 h^−1^		62.96^ab^	62.94^ab^	65.71^a^	57.13^b^	57.03^b^	2.21	0.03
Degradability at 0.08 h^−1^		52.84^a^	52.86^a^	56.39^a^	46.94^b^	46.61^b^	2.30	0.04
Potential degradability		99.70	98.81	97.32	98.79	99.95	1.49	0.53

^1^T1 (MON during the entire feeding period); T2 (VM during the entire feeding period); T3 (MON + VM during the adaptation period and only VM during the finishing period 1 and 2); T4 (MON + VM during the entire feeding period); T5 (MON + VM during the adaptation and finishing period 1 and only VM during the finishing period 2). ^2^SEM: standard error of the mean, referent to *n* = 6 pens per treatment; Values within a row with different lower case letters differ (*p* < 0.05).

Crude protein degradability data are presented in Table 4. No differences were found in the finishing period 1 for crude protein degradability, as well as for potential degradability (*p* > 0.05). However, the withdrawal of MON in the finishing period 2 (T5) improved the crude protein degradability at passage rate of 2 h^−1^ in relation to the other treatments (*p* < 0.01), and the animals fed T1 and T2 had the lowest degradability at 2 h^−1^. Moreover, animals fed T1 and T5 during finishing period 2 had higher crude protein degradability at 5 h^−1^ when compared to animals receiving T2 (*p* = 0.01). Similarly, cattle fed T3 and T5 had higher degradability at 8 h^−1^ when compared to animals receiving T2 (*p* = 0.01). However, no differences were found in the finishing period 2 for potential degradability (*p* = 0.07).

**Table 4:** *In situ* degradability of crude protein for rumen cannulated cattle fed high-concentrate diets containing different combinations of virginiamycin (VM) and monensin (MON) during the feedlot period.

Item^2^	Period	Treatments^1^					SEM^2^	*p*-value
	Adaptation:	MON	VM	MONVM	MONVM	MONVM		
	Finishing 1:	MON	VM	VM	MONVM	MONVM		
	Finishing 2:	MON	VM	VM	MONVM	VM		
		(T1)	(T2)	(T3)	(T4)	(T5)		
*Finishing period 1*								
Degradability at 0.02 h^−1^		66.09	64.43	64.17	64.21	66.03	3.21	0.94
Degradability at 0.05 h^−1^		49.16	48.04	46.15	48.84	50.52	2.94	0.74
Degradability at 0.08 h^−1^		24.75	18.03	16.37	17.44	18.02	2.29	0.06
Potential degradability		97.08	91.97	98.00	90.08	91.66	2.39	0.10
*Finishing period 2*								
Degradability at 0.02 h^−1^		61.70^d^	61.93^d^	68.77^c^	71.23^b^	75.71^a^	1.06	<0.01
Degradability at 0.05 h^−1^		19.64^a^	15.52^b^	18.22^ab^	17.34^ab^	19.34^a^	1.76	0.01
Degradability at 0.08 h^−1^		17.95^ab^	15.52^b^	18.22^a^	17.36^ab^	19.34^a^	1.45	0.01
Potential degradability		98.86	99.28	96.19	95.31	92.83	1.84	0.07

^1^T1 (MON during the entire feeding period); T2 (VM during the entire feeding period); T3 (MON + VM during the adaptation period and only VM during the finishing period 1 and 2); T4 (MON + VM during the entire feeding period); T5 (MON + VM during the adaptation and finishing period 1 and only VM during the finishing period 2). ^2^SEM: standard error of the mean, referent to *n* = 6 pens per treatment; Values within a row with different lower case letters differ (*p* < 0.05).

### 3.2 Feeding behavior and particle sorting

The feeding behavior data for the finishing period 1 are presented in Table 5. Differences were found only for time spent resting (*p* = 0.04) and DMI (p = 0.04). The lowest values observed for time spent resting were found in animals fed T2 and T3 when compared to animals receiving T5. In addition, cattle fed T1 had lower DMI when compared to T2 and T3, where the withdrawal of MON at the end of the adaptation period showed an increase in DMI. Moreover, animals fed T4 sorted (*p* < 0.01) more intensively for short particles when compared to animals receiving T2 and T3. In addition, cattle fed T4 sorted (*p* = 0.03) against fine particles when compared to animals receiving T2 and T3.

**Table 5:** Feeding behavior and particle sorting for rumen cannulated cattle fed high-concentrate diets containing different combinations of virginiamycin (VM) and monensin (MON) at finishing period 1 of the feedlot period.

Item^2^	Period	Treatments^1^					SEM^3^	*p*-value
	Adaptation:	MON	VM	MONVM	MONVM	MONVM		
	Finishing 1:	MON	VM	VM	MONVM	MONVM		
	Finishing 2:	MON	VM	VM	MONVM	VM		
		(T1)	(T2)	(T3)	(T4)	(T5)		
*Feeding behavior*								
Time spent resting, min		896.00^ab^	824.00^b^	823.00^b^	896.00^ab^	926.00^a^	38.88	0.04
Time spent ruminating, min		366.00	408.00	419.00	351.00	317.00	28.29	0.06
Time spent eating, min		178.00	208.00	198.00	193.00	197.00	22.86	0.14
DMI, Kg		10.47^c^	14.67^a^	13.38^ab^	11.89^bc^	11.91^bc^	1.18	0.04
Meal length, min		19.55	16.35	14.57	17.82	16.25	2.73	0.32
Meals per day, n		10.40	13.20	13.80	11.80	13.20	1.58	0.06
DMI per meal, Kg		1.44	1.14	1.05	1.13	1.04	0.19	0.21
RR of DM, min/kg de DM		33.09	28.91	31.68	30.85	26.97	3.35	0.43
ER of DM, min/kg de DM		15.49	14.95	15.03	16.17	16.71	2.04	0.20
NDF intake		3.97	5.00	4.35	4.06	4.12	0.44	0.47
ER of NDF, min/kg de DM		51.76	42.93	45.22	48.21	48.24	5.96	0.79
RR of NDF, min/kg de DM		108.73	83.17	104.02	86.28	76.27	12.22	0.29
*Particle sorting*^4^								
Long		1.00	0.95	0.97	1.06	1.06	0.08	0.37
Medium		1.04	1.01	1.02	1.11	1.06	0.03	0.07
Short		1.03^ab^	1.02^b^	1.02^b^	1.04^a^	1.03^ab^	0.01	<0.01
Fine		0.95^ab^	0.97^a^	0.97^a^	0.92^b^	0.94^ab^	0.02	0.03

^1^T1 (MON during the entire feeding period); T2 (VM during the entire feeding period); T3 (MON + VM during the adaptation period and only VM during the finishing period 1 and 2); T4 (MON + VM during the entire feeding period); T5 (MON + VM during the adaptation and finishing period 1 and only VM during the finishing period 2). ^2^DMI, dry matter intake; ER, eating rate; DM, dry matter; RR, rumination rate; NDF, neutral detergent fiber. ^3^SEM, standard error of the mean, referent to *n* = 6 pens per treatment. Values within a row with different lower case letters differ (*p* < 0.05). ^4^Particle fraction retained on screens of 19 mm (long), 8 mm (medium), 1.18 mm (short) and a pan (fine).

Regarding the finishing period 2, animals fed T2 and T3 had greater DMI (*p* = 0.02) when compared to animals receiving other treatments, where the treatment T5 showed the lowest value (Table 6). Similarly, cattle fed T3 had greater DMI per meal (*p* = 0.04) when compared to animals fed T1, T4 and T5. In addition, for animals fed T1 had higher eating rate of NDF (p = 0.04) when compared to other treatments.

**Table 6:** Feeding behavior and particle sorting for rumen cannulated cattle fed high-concentrate diets containing different combinations of virginiamycin (VM) and monensin (MON) at finishing period 2 of the feedlot period.

Item^2^	Period	Treatments^1^					SEM^3^	*p*-value
	Adaptation:	MON	VM	MONVM	MONVM	MONVM		
	Finishing 1:	MON	VM	VM	MONVM	MONVM		
	Finishing 2:	MON	VM	VM	MONVM	VM		
		(T1)	(T2)	(T3)	(T4)	(T5)		
*Feeding behavior*								
Time spent resting, min		908.00	901.00	885.00	906.00	922.00	46.49	0.93
Time spent ruminating, min		315.00	356.00	367.00	331.00	322.00	41.64	0.73
Time spent eating, min		217.00	183.00	188.00	203.00	196.00	24.96	0.40
DMI, Kg		11.98^b^	13.49^a^	14.11^a^	12.04^b^	10.86^c^	0.76	0.02
Meal length, min		18.31	17.59	18.38	15.81	18.81	2.18	0.75
Meals per day, n		13.00	11.00	10.60	13.00	12.80	2.15	0.15
DMI per meal, Kg		1.14^b^	1.41^ab^	1.60^a^	1.10^b^	1.20^b^	0.30	0.04
RR of DM, min/kg de DM		27.15	27.10	26.04	27.45	28.92	3.30	0.96
ER of DM, min/kg de DM		18.73	13.78	13.47	16.67	19.56	2.47	0.06
NDF intake		2.72	3.72	3.51	3.58	3.16	0.34	0.14
ER of NDF, min/kg de DM		90.60^a^	51.95^b^	54.11^b^	57.90^b^	64.92^b^	11.61	0.04
RR of NDF, min/kg de DM		134.76	108.60	104.61	97.82	98.16	19.82	0.53
*Particle sorting*								
Long		1.17	0.89	1.06	1.08	1.06	0.08	0.14
Medium		1.11	0.97	1.05	1.11	1.07	0.04	0.15
Short		1.03	1.04	1.03	1.04	1.02	0.01	0.40
Fine		0.93	0.99	1.02	0.91	0.96	0.04	0.19

^1^T1 (MON during the entire feeding period); T2 (VM during the entire feeding period); T3 (MON + VM during the adaptation period and only VM during the finishing period 1 and 2); T4 (MON + VM during the entire feeding period); T5 (MON + VM during the adaptation and finishing period 1 and only VM during the finishing period 2). ^2^DMI, dry matter intake; ER, eating rate; DM, dry matter; RR, rumination rate; NDF, neutral detergent fiber. ^3^SEM, standard error of the mean, referent to *n* = 6 pens per treatment. Values within a row with different lower case letters differ (*p* < 0.05). ^4^Particle fraction retained on screens of 19 mm (long), 8 mm (medium), 1.18 mm (short) and a pan (fine).

### 3.3 Dry matter intake and ruminal pH

The results of DMI and ruminal pH are presented in Table 7. Animals fed T2 in finishing period 1 had greater DMI (Table 7) when compared to other treatments, and also animals fed T3 had greater DMI when compared to animals receiving T1 (*p* < 0.01). For rumen pH variables in the finishing period 1, cattle fed T3 had greater mean pH when compared to the animals fed T1 and T5 (*p* = 0.02). Moreover, the treatments T2 and T3 reduced the duration of time in which pH was below 6.2 (*p* < 0.01) when compared to the other treatments. Likewise, area under the curve for ruminal pH 6.2 decreased in animals fed T2 and T3 when compared to the other treatments (*p* = 0.02). In addition, the ruminal temperature was higher for the animals fed T2 and T3 when compared to animals receiving T1 and T5 in finishing period 1 (*p* < 0.01).

**Table 7:** Ruminal pH of rumen cannulated cattle fed high-concentrate diets containing different combinations of virginiamycin (VM), and monensin (MON) at different stages of the feedlot.

Item^2^	Period	Treatments^1^					SEM^3^	*p*-value
	Adaptation:	MON	VM	MONVM	MONVM	MONVM		
	Finishing 1:	MON	VM	VM	MONVM	MONVM		
	Finishing 2:	MON	VM	VM	MONVM	VM		
		(T1)	(T2)	(T3)	(T4)	(T5)		
*Finishing period 1*								
DMI, kg		11.50^c^	14.80^a^	13.60^b^	12.50^bc^	12.50^bc^	0.78	<0.01
Mean pH		6.00^bc^	6.17^ab^	6.24^a^	6.03^abc^	5.86^c^	0.13	0.02
Maximum pH		6.80	6.89	6.84	6.76	6.65	0.08	0.21
Minimum pH		5.27	5.20	5.36	5.40	5.16	0.10	0.29
Duration pH <5.2, h		198.00	144.00	4.00	222.00	700.00	307.68	0.52
Duration pH <5.6, h		936.00	414.00	400.00	612.00	1190.00	434.45	0.29
Duration pH <6.2 h		2588.00^a^	1982.00^b^	1700.00^b^	2518.00^a^	2970.00^a^	339.08	<0.01
Area pH < 5.2 pH x h		27.17	28.82	0.09	170.05	229.30	115.79	0.47
Area pH < 5.6 pH x h		251.78	124.45	62.84	324.49	578.69	259.73	0.70
Area pH < 6.2 pH x h		1293.99^ab^	780.89^bc^	642.31^c^	1206.05^ab^	1781.05^a^	479.60	0.02
Temperature		39.09^c^	39.34^a^	39.39^a^	39.25^ab^	39.16^bc^	0.11	<0.01
Ox-redox potential		−353.21	−350.39	−360.34	−349.59	−352.04	19.96	0.91
*Finishing period 2*								
DMI, kg		11.00^b^	12.20^a^	12.30^a^	10.60^b^	11.00^b^	1.01	<0.01
Mean pH		6.06	6.20	5.88	6.07	5.92	0.15	0.12
Maximum pH		6.83^b^	6.86^b^	6.86^b^	6.96^b^	7.30^a^	0.15	0.03
Minimum pH		5.25^a^	5.41^a^	4.84^c^	5.19^ab^	5.07^b^	0.08	<0.01
Duration pH <5.2, h		114.00^bc^	0.00^c^	892.00^a^	514.00^ab^	284.00^ab^	343.46	<0.01
Duration pH <5.6, h		780.00^ab^	158.00^b^	1492.00^a^	1138.00^a^	1378.00^a^	492.75	0.03
Duration pH <6.2 h		2476.00	1982.00	2646.00	2290.00	2928.00	437.17	0.10
Area pH < 5.2 pH x h		8.30^bc^	0.00^c^	269.71^a^	46.57^b^	24.89^b^	87.08	0.01
Area pH < 5.6 pH x h		165.25^c^	13.63^d^	737.31^a^	409.78^b^	345.94^b^	251.58	<0.01
Area pH < 6.2 pH x h		1116.80^ab^	565.29^b^	1947.51^a^	1408.75^ab^	1638.84^a^	514.30	0.04
Temperature		39.07^c^	39.17^bc^	39.43^a^	39.33^ab^	39.40^a^	0.07	<0.01
Ox-redox potential		−360.78	−354.91	−356.57	−342.03	−337.61	18.12	0.19

^1^T1 (MON during the entire feeding period); T2 (VM during the entire feeding period); T3 (MON + VM during the adaptation period and only VM during the finishing period 1 and 2); T4 (MON + VM during the entire feeding period); T5 (MON + VM during the adaptation and finishing period 1 and only VM during the finishing period 2). ^2^DMI, dry matter intake. ^3^SEM, standard error of the mean, referent to *n* = 6 pens per treatment. Values within a row with different lower case letters differ (*p* < 0.05).

For the finishing period 2, animals fed T2 and T3 in finishing period 1 had greater DMI (p < 0.01; Table 7) when compared to animals receiving other treatments. In addition, cattle fed T5 had higher maximum pH (*p* = 0.03) in relation to the other treatments, while the minimum pH was lower for the animals fed T3 and T5 when compared other treatments (*p* < 0.01). Moreover, the duration of time in which pH was below 5.2 was longer in the cattle fed T3 (*p* < 0.01) when compared to animals receiving T1 and T2. Similarly, animals fed T3, T4 and T5 had increased the duration of time in which pH was below 5.6 (p = 0.03) when compared to other treatments during finishing period 2. The area under the curve for ruminal pH 5.2 was higher for the animals fed T3 (*p* = 0.01) when compared to T1 and T2. Moreover, the area under the curve for ruminal pH 5.6 was higher for the animals fed T3 (*p* = 0.01) when compared to T2, and the area under pH 6.2 was higher for the animals fed T3 and T5 (*p* < 0.01) when compared to animals receiving T2. Likewise, the ruminal temperature was higher for the animals fed T3 and T5 when compared to animals receiving T1 and T2 in finishing period 2 (*p* < 0.01).

### 3.4 Ruminal fermentation and protozoa counting

**Figure 1:**
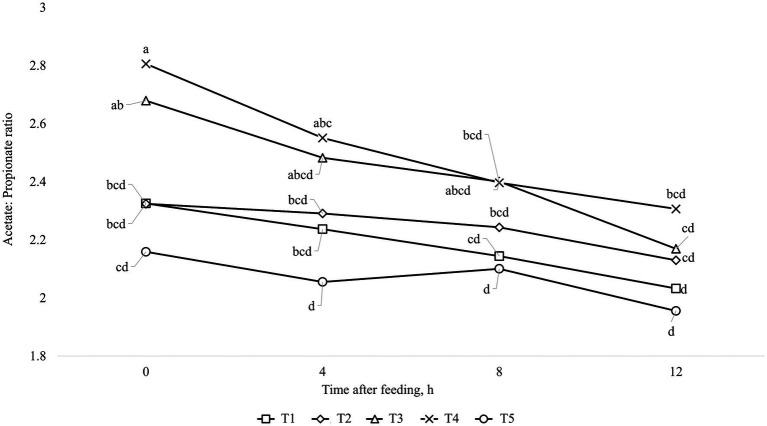
Interaction between treatment and time after feeding about the levels of Acetate: Propionate, in animals fed with MON during the entire feeding period (■; T1); VM during the entire feeding period (♦; T2); VM + MON during the adaptation and only VM during the finishing period 1 and 2 (▲; T3); VM + MON during the entire feeding period (**x;** T4); VM + MON during the adaptation and finishing period 1 and only VM during the finishing period 2 (●; T5) on Nellore yearling bulls fed high-concentrate diets. Means without a common letter differ (*p* < 0.05).

**Figure 2:**
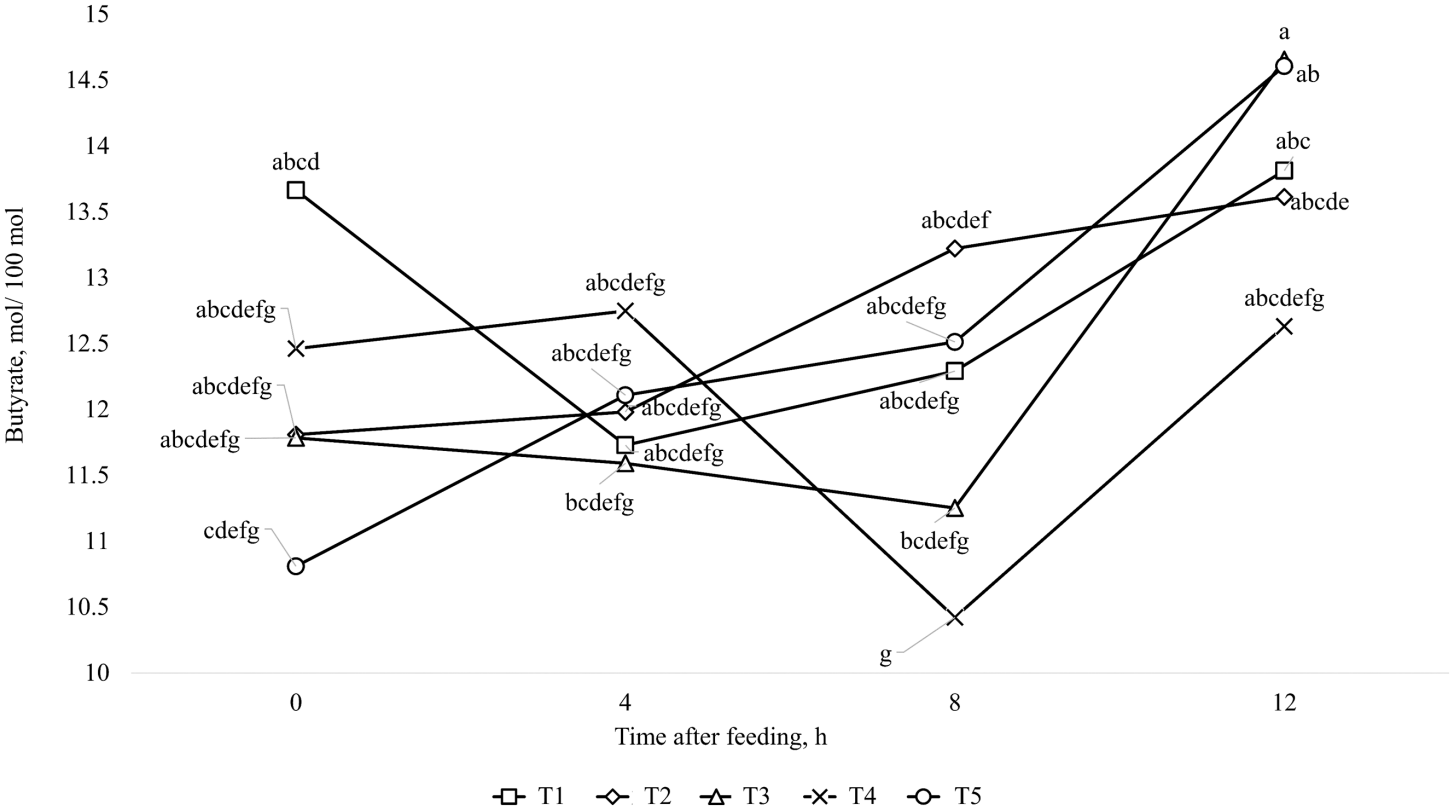
Interaction between treatment and time after feeding about the levels of Butyrate, in animals fed with MON during the entire feeding period (■; T1); VM during the entire feeding period (♦; T2); VM + MON during the adaptation and only VM during the finishing period 1 and 2 (▲; T3); VM + MON during the entire feeding period (**x;** T4); VM + MON during the adaptation and finishing period 1 and only VM during the finishing period 2 (●; T5) on Nellore yearling bulls fed high-concentrate diets. Means without a common letter differ (*p* < 0.05).

The results of ruminal fermentation products are presented in Table 8. There was no significant treatment effect (*p* > 0.05) for any of the ruminal fermentation parameters in both finishing periods 1 and 2. However, time effects were found in some of the measured variables. Regarding finishing period 1, the concentration of acetate and butyrate were affected quadratically (*p* < 0.01) by time after feeding, where the lowest value was found at 4 h after feeding. Furthermore, the concentration of propionate and total SCFA were linearly increased (*p* < 0.01) by time after feeding. Moreover, it was observed an interaction between treatments and time for Acetate: Propionate ratio (*p* = 0.03), which is shown in Figure 1. For the finishing period 2, the concentration of acetate, propionate, and total SCFA were linearly increased (*p* < 0.01), while for the levels of ruminal NH_3_-N and for Acetate: Propionate ratio were linearly decreased (*p* < 0.01) by time after feeding. In addition, it was observed an interaction between treatments and time for concentration of butyrate (*p* = 0.03), which is shown in Figure 2.

**Table 8:** Evaluation of ruminal fermentation products of rumen cannulated cattle fed high concentrate diets containing combined use of virginiamycin (VM), and monensin (MON) at different stages of the feedlot.

Item^2^	Period	Treatments^1^					Time after Feeding (h)					*p*-value		
	Adaptation:	MON	VM	MONVM	MONVM	MONVM	0	4	8	12	SEM^2^	Trat	Time	Trat*Time
	Finishing 1:	MON	VM	VM	MONVM	MONVM								
	Finishing 2:	MON	VM	VM	MONVM	VM								
		(T1)	(T2)	(T3)	(T4)	(T5)								
*Finishing period 1*														
Acetate, mol/ 100 mol		62.94	66.37	67.71	65.73	62.05	62.04	61.72	64.80	71.28	2.34	0.40	<0.01(Q)	0.36
Propionate, mol/ 100 mol		30.24	31.23	28.55	26.95	31.23	26.21	27.77	29.91	34.67	2.16	0.24	<0.01(L)	0.30
Butyrate, mol/ 100 mol		13.81	13.88	14.41	14.11	12.78	14.13	12.74	13.47	14.86	0.75	0.76	<0.01(Q)	0.20
Total SCFA, mM		106.98	111.48	110.66	106.78	106.06	102.37	102.22	108.18	120.81	3.76	0.66	<0.01(L)	0.15
Acetate: Propionate		2.19	2.25	2.43	2.52	2.07	2.46	2.32	2.26	2.12	0.18	0.02	<0.01	0.03
Lactate mM		0.023	0.027	0.018	0.017	0.019	0.019	0.023	0.020	0.021	0.01	0.09	0.83	0.46
NH_3_-N mg/ dl		21.23	20.29	17.66	19.61	17.47	19.68	16.87	20.45	20.01	1.99	0.33	0.18	0.16
*Finishing period 2*														
Acetate, mol/ 100 mol		62.01	64.45	63.81	60.36	60.84	55.18	60.20	61.14	72.66	2.29	0.60	<0.01(L)	0.26
Propionate, mol/ 100 mol		31.82	27.05	32.67	31.10	32.79	25.25	28.85	30.04	40.19	2.63	0.47	<0.01(L)	0.44
Butyrate, mol/ 100 mol		12.88	12.66	12.32	12.07	12.51	12.11	12.03	11.94	13.86	1.15	0.83	<0.01	<0.01
Total SCFA, mM		106.70	104.15	108.80	103.52	106.14	92.53	101.08	103.12	126.71	4.84	0.82	<0.01(L)	0.12
Acetate: Propionate		2.00	2.49	2.12	2.05	1.96	2.26	2.19	2.14	1.92	0.14	0.26	<0.01(L)	0.45
Lactate mM		0.021	0.021	0.016	0.020	0.026	0.021	0.020	0.016	0.027	0.01	0.19	0.56	0.12
NH_3_-N mg/ dl		14.92	13.31	15.34	15.75	9.00	17.48	15.41	10.88	10.88	2.10	0.06	0.04(L)	0.19

^1^T1 (MON during the entire feeding period); T2 (VM during the entire feeding period); T3 (MON + VM during the adaptation period and only VM during the finishing period 1 and 2); T4 (MON + VM during the entire feeding period); T5 (MON + VM during the adaptation and finishing period 1 and only VM during the finishing period 2). ^2^SEM, standard error of the mean, referent to *n* = 6 pens per treatment. Values within a row with different lower case letters differ (*p* < 0.05).

The results of differential counting of protozoa are presented in Table 9. Regarding the finishing period 1, animals fed T4 had greater population of protozoa from the genus *Diplodinium* (*p* = 0.04) when compared to those from animals fed T2, T3 and T5. For finishing period 2, cattle receiving T1 had greater population of protozoa from the genus *Isotricha* (*p* < 0.01), as well as greater total count (*p* = 0.04) when compared to other treatments.

**Table 9:** Differential protozoan counting of rumen cannulated cattle fed high concentrate diets containing combined use of virginiamycin (VM), and monensin (MON) at different stages of the feedlot.

Item	Period	Treatments^1^					SEM^2^	*p*-value
	Adaptation:	MON	VM	MONVM	MONVM	MONVM		
	Finishing 1:	MON	VM	VM	MONVM	MONVM		
	Finishing 2:	MON	VM	VM	MONVM	VM		
		(T1)	(T2)	(T3)	(T4)	(T5)		
*Finishing period 1*								
*Dasytricha* x 10^3^/ ml		8.88	6.24	8.88	9.36	6.24	1.20	0.19
*Isotricha* x 10^3^/ ml		1.92	1.44	2.16	2.40	1.44	0.56	0.68
*Entodinium* x 10^3^/ ml		74.64	55.20	62.16	63.12	56.64	4.84	0.07
*Diplodinium* x 10^3^/ ml		19.68^ab^	15.60^bc^	12.24^c^	21.12^a^	15.84^bc^	2.05	0.04
Total x 10^3^ / ml		105.12	78.48	85.44	96.00	80.16	7.65	0.10
*Finishing period 2*								
*Dasytricha* x 10^3^/ ml		10.08	7.44	7.92	6.96	6.96	1.32	0.45
*Isotricha* x 10^3^/ ml		3.84^a^	1.44^bc^	0.96^c^	2.40^b^	0.72^c^	0.57	<0.01
*Entodinium* x 10^3^/ ml		64.32	59.04	56.16	53.28	60.72	3.44	0.06
*Diplodinium* x 10^3^/ ml		21.60	14.16	16.08	16.08	16.08	2.08	0.16
Total x 10^3^ / ml		99.84^a^	82.08^b^	81.12^b^	78.72^b^	84.48^ab^	4.99	0.04

^1^T1 (MON during the entire feeding period); T2 (VM during the entire feeding period); T3 (MON + VM during the adaptation period and only VM during the finishing period 1 and 2); T4 (MON + VM during the entire feeding period); T5 (MON + VM during the adaptation and finishing period 1 and only VM during the finishing period 2). ^2^SEM, standard error of the mean, referent to n = 6 pens per treatment. Values within a row with different lower case letters differ (*p* < 0.05).

### 3.5 Ruminal dynamics

The results of ruminal dynamics are presented in Table 10. There was no significant treatment effect for most of the ruminal dynamics variables evaluated (*p* > 0.05). However, animals fed T2 and T3 had higher disappearance rate, expressed in kg/h (*p* = 0.03), when compared to animals receiving T1 and T4.

**Table 10:** Ruminal dynamics of rumen cannulated cattle fed high concentrate diets containing combined use of virginiamycin (VM), and monensin (MON) at different stages of the feedlot.

Item^2^	Period	Treatments^1^					SEM^3^	*p*-value
	Adaptation:	MON	VM	MONVM	MONVM	MONVM		
	Finishing 1:	MON	VM	VM	MONVM	MONVM		
	Finishing 2:	MON	VM	VM	MONVM	VM		
		(T1)	(T2)	(T3)	(T4)	(T5)		
Total net mass, Kg		45.65	46.59	46.29	45.77	45.26	1.99	0.88
Total solid mass, Kg		7.47	8.51	8.51	8.00	7.94	0.49	0.07
Total mass, Kg		53.11	55.09	54.80	53,76	53.20	2.37	0.49
Total net mass, % BW		7.19	7.17	7.24	7.24	7.21	0.40	0.99
Total solid mass, % BW		1.17	1.30	1.33	1.27	1.27	0.08	0.20
Total mass, % BW		8.36	8.47	8.57	8.50	8.48	0.47	0.91
Kt, Kg/h		0.48^b^	0.58^a^	0.57^a^	0.49^b^	0.54^ab^	0.03	0.03
Kt, %/ h		6.56	6.96	6.74	6.29	7.16	0.61	0.52
DP^2^ of Ruminal content, %		14.09	15.39	15.56	14.86	14.83	0.55	0.38

^1^T1 (MON during the entire feeding period); T2 (VM during the entire feeding period); T3 (MON + VM during the adaptation period and only VM during the finishing period 1 and 2); T4 (MON + VM during the entire feeding period); T5 (MON + VM during the adaptation and finishing period 1 and only VM during the finishing period 2). ^2^DP, Dried pasta; Kt, disappearance rate. ^3^SEM, standard error of the mean, referent to *n* = 6 pens per treatment. Values within a row with different lower case letters differ (*p* < 0.05).

## 4 Discussion

The transition of animals into a feedlot environment triggers significant metabolic changes, primarily driven by the shift from a forage-based diet to one rich in fermentable ingredients. This transition holds paramount importance within the overall production cycle. Consequently, the utilization of products designed to modulate rumen fermentation processes and mitigate undesirable effects has become widespread. In our study, we employed two specific molecules, Sodium Monensin and Virginiamycin, either alone or in combination at full doses (30 mg/kg and 25 mg/kg, respectively). We designed our experiment to remove one of these molecules at two critical junctures: at the end of the adaptation phase (when animals transition to the finishing diet), and after an increase in the energy density of the finishing diet (achieved through elevated concentrate inclusion).

In this scenario, assessing DMI serves as a crucial metric to evaluate how well cattle are either accepting or adapted to the diets (45), and a quicker attainment of a DMI equivalent to 2% of BW indicates superior diet adaptation. Rigueiro et al. (10) noted that Nellore bulls fed only VM during the adaptation phase reached a DMI of 2% of their initial BW within an average of 4.3 days, whereas those fed MON took 20.7 days to achieve a similar intake. Monensin, the most commonly used molecule in feedlots (2, 3), functions as a regulator of animal DMI, as corroborated by studies (7). Consequently, the removal of MON, particularly at the end of the adaptation period and during the final phase of the feedlot, can enhance animal consumption. This phenomenon became apparent in our study when MON was withdrawn at the end of the adaptation phase. In this instance, animals exhibited increased DMI compared to those consuming MON alone, and during the finishing diet 2, these animals showed a higher DMI compared to other treatments involving the combination of additives. Similar observations were reported by Rigueiro et al. (10) in performance animals. However, the withdrawal of MON, combined with an increase in energy density in the finishing diet, did not affect DMI in our study. Squizatti et al. (24) reported that the addition of MON in feedlot diets, whether with or without VM, leads to a reduction in DMI. This reduction in intake may be attributed to prolonged ruminal retention time of dry matter, and to the increased propionic acid production, known to regulate animal satiety in ruminants (46).

Concerning metabolic data, the removal of MON after the adaptation period improved the potential degradability of dry matter compared to other treatments involving additive combinations or VM alone during the first finishing phase. However, during the second finishing phase, the use of additive combinations, either throughout the entire period or with MON removal in this phase, reduced starch degradability at passage rates of 2, 5, and 8 h^−1^ compared to MON removal at the beginning of the adaptation phase. This suggests that the prolonged use of additive combinations may hinder the utilization of one of the primary components of feedlot diets (2, 3). Reduced starch degradability in the rumen results in more starch passing into the intestines, which can reduce diet energy utilization (47, 48), and trigger inflammatory processes in animals (49), thus impacting animal performance (10). Moreover, Squizatti et al. (24) also reported that cattle fed MON decreased ruminal degradability of DM, NDF, ADF, starch, NFE, and TDN, but increased CP degradability when compared to animals consuming VM.

Protein degradability data only exhibited an effect during the second finishing phase, where VM inclusion in diets appeared to reduce protein degradability. However, the combined use of additives, regardless of the phase, seemed to mitigate this effect. This aligns with Ives et al. (15), who reported that VM had a protein-sparing effect on diet proteins in the rumen of steers fed corn-based finishing diets, and this effect can potentially enhance the metabolizable protein supply to cattle due to the observed increase in the concentration of α-amino nitrogen in the rumen for the VM-based diets. Similarly, *In vitro* studies by Van Nevel et al. (16) demonstrated that VM reduced deamination and casein degradation. Additionally, the inclusion of ionophore can also decrease CP degradation in the rumen, leading to an increase in the amount of protein bypassing the rumen (50). In addition to the improvement in energy efficiency, MON can reduce proteolysis and deamination in the rumen (51, 52). Thus, this ionophore reduces losses of proteins and amino acids that would be degraded and converted into ammonia (53), resulting in greater passage of dietary proteins and amino acids to the small intestine (54). However, other studies have reported an increase in protein degradability in animals fed ionophores (55, 56). Mazza et al. (56) reported an increase in CP digestibility in animals receiving MON. Furthermore, the same authors observed that increasing the proportion of concentrates in the diet linearly increased the apparent digestibility of crude protein compared to a diet with more roughage. In the present study, animals fed T1 showed greater protein degradability during the second finishing phase. One potential explanation could be attributed to the effects of MON on specific groups of bacteria in the ruminal environment, considering that certain Gram-negative bacteria in the rumen are sensitive to ionophore initially, and there is potential for adaptation even among Gram-positive bacteria (57). Moreover, recent studies have also reported a residual and long-term effect of ionophores on ruminal fermentation parameters and ionophores-insensitive microbe population after withdrawal from the diet (58–60). *Streptococcus bovis*, a Gram-positive bacterium, is widely recognized as one of the primary bacterial species involved in ruminal proteolysis due to high peptidolytic activity, particularly in leucine aminopeptidase (61, 62). However, research indicates that proteolytic activity in the rumen may be attributed to several associated species or potentially to complementary functions. Hence, it’s challenging to solely attribute specific stages of proteolysis to the activity of a single bacterium, as these species may employ complementary approaches in their proteolytic activities (61–63). Dennis et al. (64) reported that out of the three strains of *S. bovis* tested (*S. bovis* 124), two were susceptible to MON, whereas the third strain exhibited resistance to it. The authors highlighted this resistant strain of *S. bovis* as an exception to the general notion that monensin inhibits gram-positive bacteria (65–67). In this context, monensin-resistant bacteria might be found in greater numbers in rumens of animals fed MON when compared to those not fed MON (65, 68), and this could result in higher protein degradability, as observed in the present study.

Notably, while dry matter degradability during the first finishing phase was higher with MON removal, pH was higher when MON was removed (T3) compared with MON (T1) and the combination of MON+VM (T5), even with greater DMI. Moreover, the duration of pH below 6.2 and the area under the pH curve below 6.2 were lower when MON was removed after the adaptation period, in comparison to animals consuming MON or other additive combinations. This implies that the presence of MON tends to increase the consumption of shorter feed particles compared to VM or MON removal at first finishing period. In the second finishing phase, no differences were observed in particle selection in response to the treatments. However, concerning feeding behavior, animals fed T1 showed higher ERNDF compared to the other treatments. The justification for this increase is related to the low consumption of NDF and the increase in TSE for animals fed T1. In addition to the effects on ruminal fermentation, changes also occur in the satiety mechanism in response to MON, which may result in a reduction in the amount of food ingested, an increase in meal frequency, a reduction in meal size, and an increase in the number of daily meals, thus dividing the entry of passive fermentation substrate. MON enhances propionic acid production, increasing the supply of propionate to the liver and hepatic gluconeogenic flux. This increased oxidation in the liver triggers a signal of satiety, leading to a reduction in meal size (69). This may be one of the points that helps minimize the decrease in ruminal pH (70). Erickson et al. (71) observed that steers supplemented with MON visited the feed bunk more frequently and consumed less during each meal compared to the control group. In this context, MON in the present study affected feeding behavior, increasing the number of meals per day in animals fed T1. Consequently, this change in feeding behavior results in pH changes, where animals fed T1 showed higher minimum pH, less duration of time in which pH was below pH5.2, and smaller area under the curve for ruminal pH < 5.2. Additionally, the ruminal temperature of animals fed T1 was low during the finishing phase, precisely the group that showed the lowest DMI.

Interestingly, rumen temperature was higher in treatments involving VM. The animals fed with T2, T3, and T4 during finishing phase 1, and T3, T4, and T5 during finishing phase 2, exhibited higher rumen temperature values. Notably, these treatments excluded the use of MON alone. Additionally, treatments T3, T4, and T5 showed higher values for the duration of pH below 5.6. The elevated rumen temperature and prolonged pH values below 5.6 in these treatments can be attributed to energy spilling in the rumen. Rumen microbes display inefficient cellular protein production, partly because they do not allocate all ATP exclusively for growth (72). Instead, ATP not utilized for growth is redirected towards non-growth functions like maintenance, energy storage, and energy spilling. This process, described as a futile cycle dissipating heat, involves the dissipation of energy as heat when ATP exceeds the requirements for growth, maintenance, and storage (73, 74). Both energy spilling and the synthesis of reserve carbohydrates primarily happen when there is an excess of available carbohydrates. In the rumen, animals fed grain, especially those transitioning to a high-concentrate diet, as observed in feedlot diets, have a higher abundance of carbohydrates (72). Thus, research studies have explored ways to mitigate the risk of ruminal acidosis. But, it’s crucial to note that a low pH does not necessarily lead to diminished performance (10, 23, 75, 76).

In this context, the pursuit of higher animal performance in the finishing phase implies an increase in the challenge and risks to ruminal fermentation due to the higher energy density of the diet. In the present study, we can infer that the higher energy availability and ATP resulting from VM contribute to the elevation of ruminal temperature, as well as increasing the duration and area under pH 5.2 and pH 5.6 for T3 and T4 when compared to animals fed T1 and T2 due to higher rumen fermentation. However, in the current study, the simultaneous use of MON and VM molecules does not exhibit any impact on total SCFA. This lack of effect may be attributed to the mechanism of SCFA absorption in the rumen. The regulation of ruminal pH is intricate and encompasses factors influencing both SCFA production and the removal of acid from the rumen. Various factors, such as the rate of fermentation, meal size and frequency, temperature, and fermentation pathway, can influence ruminal pH based on SCFA production and absorption (76). The dynamics of rumen SCFA are influenced by both the thermal environment and feed intake. Bedford et al. (77) reported an increase in acetate absorption with decreased DMI, potentially attributed to heightened expression of rumen epithelium transporters involved in SCFA absorption. In cases of low feed intake, the rumen epithelium seems to enhance its capacity for SCFA absorption and transport, possibly as a compensatory mechanism to boost energy balance (77). This can explain the findings for animals fed T3, exhibiting higher dry matter intake (DMI) compared to T1 and T2, impacting SCFA absorption and, consequently, the area and duration at pH below 5.6. However, during the second finishing phase, combinations of additives resulted in an extended duration of pH below 5.2 and 5.6, along with larger areas under the respective pH curves, in comparison to animals consuming VM alone. This consumption pattern suggested that withdrawing MON at the end of adaptation increased DMI per meal compared to other additive combinations and MON, aligning with findings reported by Fanning et al. (78). Furthermore, as MON reduces DMI, the efficiency of neutral detergent fiber (NDF) feeding was higher in treatments using VM. It is crucial to acknowledge that as pH decreases, the proportion of undissociated SCFA increases, and only undissociated SCFA are permeable across the lipid bilayer of cells. Consequently, a pH reduction would enhance the proportion of undissociated SCFA, allowing them to freely diffuse across the rumen epithelium (76, 79). This phenomenon elucidates the limited enhancement observed in treatment on SCFA concentration and rumen fermentation parameters.

In this scenario, the rumen pH and NH_3_-N concentration play a pivotal role in influencing the end products of rumen fermentation, particularly SCFA and microbial protein, which serve as vital sources of energy and amino acids for animals. The release and proportion of SCFA in the rumen are contingent on factors such as pH (80, 81), dilution rate (82), and substrate (83), all controlled by the host and the population of microorganisms inhabiting the rumen. Therefore, antimicrobials like MON and VM help regulate these strains, particularly bacteria. Although the metabolite data in our study did not differ between treatments, it exhibited time-dependent effects, with an interaction observed for the Acetate:Propionate ratio during the first finishing phase. The breakdown of this interaction revealed that treatment involving additive combinations throughout the entire period exhibited a higher Acetate:Propionate ratio at the initial time (0 h), which decreased in the subsequent hours of collection (Figure 1). This dynamic can be attributed to the quadratic response of acetate levels, which were higher at time 0, decreased at time 4, and then rose again in subsequent hours. Conversely, propionate concentrations exhibited a linear increase, consistent with total SCFA levels. Butyrate levels also displayed a quadratic response (Figure 2), mirroring the pattern observed with acetate levels. As previously mentioned, when faced with an excess of carbohydrates, rumen microbes may respond beyond the simple processes of spilling energy and synthesizing reserve carbohydrates. Moreover, various interconversions of SCFA and their utilization for anabolism can lead anaerobic bacteria to occupy metabolite intermediates, potentially expending ATP and reducing the efficiency of microbial protein production (72). Interconversions among SCFA, such as from acetate to butyrate, can consume ATP and negatively impact the efficiency of microbial protein production. Several research studies have noted significant interconversion from acetate to butyrate, minimal interconversion between butyrate and acetate or between butyrate and propionate, and almost no interconversion between acetate and propionate (72, 84, 85). Based on this information, we can suggest that there was SCFA interconversion between acetate and butyrate in the present study, reducing the concentration of acetate and increasing the concentration of butyrate, as shown in Figures 1, 2, respectively, over time after the meal in response to treatment T3. In addition, Bedford et al. (77) reported that during periods of elevated ambient temperature, butyrate production increases, but the absorption and transport capacity to remove butyrate metabolites from the epithelium decrease.

Moreover, the interaction of butyrate levels in the finishing phase 2 may be linked to population characteristics and microbial growth. In this phase, MON withdrawal resulted in lower butyrate levels before feeding compared to the last collection hour (12 h). Although it was not measured in the present study, this effect may be attributed to the activity of *Megasphaera elsdenii* in lactate fermentation, which produces butyrate and valerate as the final products at lower pH levels (20, 80, 86). Conversely, at higher pH levels, this strain of bacteria prioritizes the production of propionate (87). Diets with high energy levels can reduce protozoan populations due to lower rumen pH or increased passage rate. It’s worth noting that VM-fed cattle had higher Kt, suggesting that *Diplodinium* protozoa were washed out of the rumen (44). The increased Kt in animals consuming VM may be linked to their higher DMI, which can accelerate passage rate, thereby influencing Kt. Furthermore, animals consuming only VM had fewer *Diplodinium* protozoa in the rumen, consistent with previous reports in the literature (88, 89). However, this reduction in protozoa count did not impact NH_3_-N concentrations, as the decrease in proteolytic and deamination enzyme activity in protozoa has been linked to this reduction (90). Frazolin and Dehority (91) reported that diets rich in energy might cause a decline in the protozoan population, possibly due to decreased rumen pH or accelerated passage rate. It’s worth noting that cattle fed with VM exhibited higher Kt, suggesting the possibility that *Diplodinium* protozoa have been flushed out of the rumen (24). The increased Kt in animals consuming VM may be linked to their higher DMI, since the animals consuming VM or with the withdrawal of MON after the adaptation period had higher DMI, which can increase the passage rate, thereby influencing the Kt.

## 5 Conclusion

Based on our previous findings (23) in a performance trail, the withdrawal of MON when associated with VM, combined with a higher energy diet, during the last 40 days of the feedlot period improved overall final BW, ADG and HCW of Nellore cattle when compared to bulls fed either MON or VM. Then, we hypothesized that withdrawing MON combined with a higher energy diet during the final third of the feedlot period improves the ruminal fermentation. In the present study, there is no substantial improvement on the rumen fermentation parameters by the concurrent utilization of MON and VM molecules. Our findings suggest that the combined use of these molecules during the adaptation phase, followed by the subsequent removal of MON, enhances the ruminal fermentation characteristics of the animals without adverse effects on ruminal metabolism. However, the higher starch and protein degradability did not translate into better rumen fermentation.

## Data availability statement

The raw data supporting the conclusions of this article will be made available by the authors, without undue reservation.

## Ethics statement

The animal study was approved by São Paulo State University Ethical Committee for Animal Research (protocol number CEUA - 154/2016). The study was conducted in accordance with the local legislation and institutional requirements.

## Author contributions

JD: Conceptualization, Data curation, Formal analysis, Investigation, Methodology, Writing – original draft. AR: Investigation, Writing – review & editing. AS: Investigation, Writing – review & editing. MP: Investigation, Writing – review & editing. LF: Investigation, Writing – review & editing. BD: Investigation, Writing – review & editing. ED: Investigation, Writing – review & editing. LS: Investigation, Writing – review & editing. DC: Investigation, Writing – review & editing. KS: Investigation, Writing – review & editing. JS: Data curation, Formal analysis, Visualization, Writing – review & editing. DM: Conceptualization, Data curation, Formal Analysis, Funding acquisition, Project administration, Supervision, Writing – original draft, Writing – review & editing.
